# The Expanded Program on Immunization in the English- and Dutch-speaking Caribbean (1977–2016): reasons for its success

**DOI:** 10.26633/RPSP.2017.127

**Published:** 2017-12-20

**Authors:** Karen N Lewis-Bell, Beryl Irons, Elizabeth Ferdinand, Laura L Jackson, J. Peter Figueroa

**Affiliations:** 1 Immunization Advisor Pan American Health Organization Kingston Jamaica Immunization Advisor, Pan American Health Organization, Kingston, Jamaica; 2 Retired Immunization Advisor Pan American Health Organization Bridgetown Barbados Retired Immunization Advisor, Pan American Health Organization, Bridgetown, Barbados.; 3 Retired National EPI Manager Ministry of Health Bridgetown Barbados Retired National EPI Manager, Ministry of Health, Bridgetown, Barbados.; 4 National EPI Manager, Department of Health, Ministry of Health National EPI Manager, Department of Health, Ministry of Health Hamilton Bermuda National EPI Manager, Department of Health, Ministry of Health, Hamilton, Bermuda.; 5 PAHO Technical Advisory Group on Vaccine-preventable Diseases University of the West Indies Kingston Jamaica Professor, Public Health, Epidemiology, HIV/AIDS and Director, DrPH Program and Chair, PAHO Technical Advisory Group on Vaccine-preventable Diseases, University of the West Indies, Kingston, Jamaica.

**Keywords:** Immunization, measles, disease eradication, Caribbean region, Inmunización, sarampión, erradicación de la enfermedad, región del Caribe, Imunização, sarampo, erradicação de doenças, região do Caribe

## Abstract

The year 2017 marks the 40^th^ year of the establishment of the Expanded Program on Immunization (EPI) by the Pan American Health Organization (PAHO), the regional office of the World Health Organization (WHO) in the Americas, the first WHO region certified as eliminating poliomyelitis (1994), measles (2016), and rubella and congenital rubella syndrome (CRS) (2015). The English- and Dutch-speaking Caribbean subregion of the Americas paved the way in eliminating these diseases. This report highlights the innovative strategies used in this subregion that helped make the EPI a success.

A review of published/unpublished reports and written and oral accounts of the experiences of Immunization Advisors and national EPI managers was conducted to identify the strategies used to strengthen the Immunization program in the subregion since its implementation by countries in 1977. The results show that these include strong collective political commitment, country-specific immunization legislation, joint use of a standard coverage monitoring chart, annual meetings of national EPI managers, collaborative development of annual national Plans of Action for Immunization, coordinated implementation of vaccination campaigns, subregional oversight of surveillance and laboratory support, a performance award system for countries, and subregional standardized templates for immunization manuals and procedural guidelines. Political will and support for immunization has been particularly strong in this subregion, where 99% of EPI costs are borne by governments. Dedicated health staff and multi-country agreement and application of strategies have led to high sustained coverage and good-quality surveillance, resulting in the absence of wild polio for 34 years, measles for 25 years, CRS for 17 years, and rubella for 15 years.

## RESUMEN

En el 2017 se celebra el cuadragésimo año de la instauración del Programa Ampliado de Inmunización (PAI) por la Organización Panamericana de la Salud (OPS), la Oficina Regional de la Organización Mundial de la Salud (OMS) para las Américas. Esta fue la primera región de la OMS que obtuvo la certificación de la eliminación de la poliomielitis (1994), el sarampión (2016) y la rubéola y el síndrome de rubéola congénita (2015). La subregión de habla inglesa y holandesa del Caribe en las Américas abrió el camino a la eliminación de estas enfermedades. En el presente artículo se destacan las estrategias innovadoras utilizadas en esta subregión que contribuyeron al éxito del PAI.

Se llevó a cabo una revisión de los informes publicados e inéditos y de los relatos escritos y orales de las experiencias de los Expertos en Inmunización y los gerentes nacionales del PAI con el objeto de determinar las estrategias utilizadas con miras a fortalecer el programa de vacunación en la subregión, desde su introducción en los países en 1977. Los resultados ponen de manifiesto que los programas comportaban un fuerte compromiso político colectivo, legislaciones en materia de inmunización propias de cada país, la utilización común de un registro gráfico normalizado de monitoreo de coberturas de vacunación, reuniones anuales de los gerentes nacionales del PAI, la elaboración conjunta de planes de acción anuales nacionales sobre vacunas, la ejecución coordinada de campañas de vacunación, la supervisión de la vigilancia y el apoyo a los laboratorios a escala subregional, un sistema de reconocimiento al desempeño de los países y plantillas subregionales normalizadas de los manuales de vacunación y los procedimientos recomendados. La voluntad política y el apoyo a la vacunación han sido muy sólidos en esta subregión, donde los gobiernos sufragan 99% de los costos del PAI. La existencia de personal sanitario dedicado y los acuerdos multinacionales y la aplicación de las estrategias permitieron alcanzar una alta cobertura de manera sostenida y una vigilancia de buena calidad, cuyo resultado fue la ausencia de poliomielitis salvaje durante 34 años, de sarampión durante 25 años, del síndrome de la rubéola congénita durante 17 años y de la rubéola durante 15 años.

## RESUMO

O ano de 2017 marca o 40º aniversário da criação do Programa Ampliado de Imunização (PAI) pela Organização Pan-Americana da Saúde (OPAS), Escritório Regional da Organização Mundial da Saúde (OMS) nas Américas, primeira Região da OMS certificada como tendo eliminado a poliomielite (1994), o sarampo (2016) e a rubéola e síndrome da rubéola congênita (2015). A sub-região das Américas constituída pelos países do Caribe de língua inglesa e holandesa abriu caminho ao eliminar essas doenças. Este relato destaca as estratégias inovadoras usadas nesta sub-região que contribuíram para tornar o PAI um programa bem-sucedido.

Foi realizada uma análise de informes publicados/inéditos e relatos orais e escritos da experiência dos assessores para assuntos de imunização e coordenadores nacionais do PAI visando identificar as estratégias aplicadas para consolidar o programa nos países da sub-região desde a sua implementação em 1977. Os resultados demonstram firme compromisso político coletivo, legislação de vacinação própria em cada país, uso conjunto de uma lista padrão para o monitoramento da cobertura, reuniões anuais dos coordenadores nacionais do PAI, desenvolvimento colaborativo de planos de ação nacionais anuais para vacinação, campanhas coordenadas de vacinação, supervisão sub-regional da vigilância e infraestrutura laboratorial, sistema de premiação dos países por bom desempenho, modelos padronizados para os manuais de vacinação e protocolos de procedimentos. A sub-região se caracteriza sobretudo pela vontade e apoio políticos para vacinação, sendo 99% do custo do PAI financiados pelos governos. Equipes de saúde diligentes, acordos entre vários países e emprego de estratégias são fatores que contribuem para elevada cobertura sustentada e vigilância de boa qualidade com a consequente não ocorrência de casos de poliomielite por vírus selvagem por 34 anos, de casos de sarampo por 25 anos, de casos de síndrome da rubéola congênita por 17 anos e de casos de rubéola por 15 anos.

The year 2017 marks the 40th year of the establishment of the Expanded Program on Immunization (EPI) (“the Immunization program”) by the Pan American Health Organization (PAHO), the regional office of the World Health Organization (WHO), in the WHO Americas region, which includes the English- and Dutch-speaking Caribbean.^a^ With strategies that include the provision of guidance and support by a technical officer at the subregional level; multi-country agreements on strategies, goals, and the timing of implementation of activities such as mass vaccination campaigns; common tools and operational guidelines; and training of workers in the health and education sectors, the English- and Dutch-speaking Caribbean’s Immunization program has long eliminated the major vaccine-preventable diseases (1). The governments in this subregion have provided nearly 100% of the cost of vaccines and supplies; 98% of the total cost of the Immunization program; and a consistent supply of data, through the annual WHO/UNICEF Joint Reporting Form on Immunization.^b^ Only one country in the subregion has received financial support from Gavi,^c^ the vaccine alliance.

Strong political will, reflected in financial, policy, and legislative support; a cadre of well-trained, competent, and committed staff; and collaboration with civil society to ensure the confidence and support of the community are essential in any health program. These characteristics are evident in the English- and Dutch-speaking Caribbean’s Immunization program and have been integral to its achievements in improving the health and well-being of the subregion’s population, including the elimination and control of vaccine-preventable diseases. This report describes the innovative strategies that have made the Immunization program in the English- and Dutch-speaking Caribbean a success.

## MATERIALS AND METHODS

A review of published/unpublished reports and written and verbal accounts of the experiences of PAHO Immunization Advisors and national EPI managers for the English- and Dutch-speaking Caribbean was conducted to document the history of the program in the subregion and the strategies used to strengthen it since its initial implementation in 1977 (2).

## BACKGROUND

The EPI was launched by WHO in 1974 and expanded to the English- and Dutch-speaking Caribbean in September 1977. By 1980, it was fully established in the Caribbean Community (CARICOM) member/associate member countries^e^ as well as Caribbean Epidemiology Centre (CAREC) member countries^e^ (3). Since 1977, a PAHO Immunization Advisor for the English- and Dutch-speaking Caribbean has provided technical oversight and guidance for the national EPI managers and coordinated the implementation of collective strategies for improving coverage and surveillance for vaccine-preventable diseases (4).

CARICOM^f^ is the main forum for achieving consensus on Caribbean issues and policies, including health. Decisions and resolutions on health, such as the elimination of diseases, are made by CARICOM’s Caucus of Health Ministers. The English- and Dutch-speaking Caribbean spans a fairly wide geographic area^g^ and comprises 25 countries, islands, and territories with populations varying in size from 4,000 to 2.7 million. 

Despite its fairly small combined population of some 7 million people, the English- and Dutch-speaking Caribbean has a strong tourism sector, hosting 17 million stopover visitors and 19 million cruise ship visitors since 2010, according to the Caribbean Tourism Organization (5). Therefore, the subregion is and continues to be at great risk for reimportation of vaccine-preventable diseases that have already been eliminated.

Nearly all countries, islands, and territories in the subregion procure vaccines and supplies through the PAHO Revolving Fund for vaccines, which was established in 1979. This pooled procurement process allows countries, especially those of the English- and Dutch-speaking Caribbean, which require relatively quantities of vaccines, to access high-quality products at the lowest common price and with a 60-day line of credit (6–8).

## STRATEGIES

Vaccination of children in the countries/territories is administered mainly by the public sector through the network of clinics that form the subregion’s well-developed primary health care services. Vaccination activities are integrated in maternal and child health services and combined with other health interventions, namely routine monitoring of growth and development, including nutrition counseling. More than 80% of infants receive vaccinations from the public health sector; private sector vaccination covers 10%–20% of each birth cohort. Various strategies are used across the subregion to maintaining high vaccination coverage for each birth cohort in the routine programs of each country/territory. In addition to clinicbased follow-up and recall mechanisms for dropouts from the Immunization program, vaccines are administered through 1) home and school visits, for older children and children who fail to respond to recall messages or 2) at outreach activities such as health fairs, during special periods such as registration for school, including nurseries and day-care facilities.

About 90% of the countries use the PAHO Revolving Fund for vaccine procurement and thus have guaranteed, sustainable access to vaccines. The relatively low cost of the vaccines procured through the Fund has facilitated the introduction of new vaccines as well as elimination activities such as mass follow-up and “catch-up” vaccination campaigns.

**FIGURE 1.**
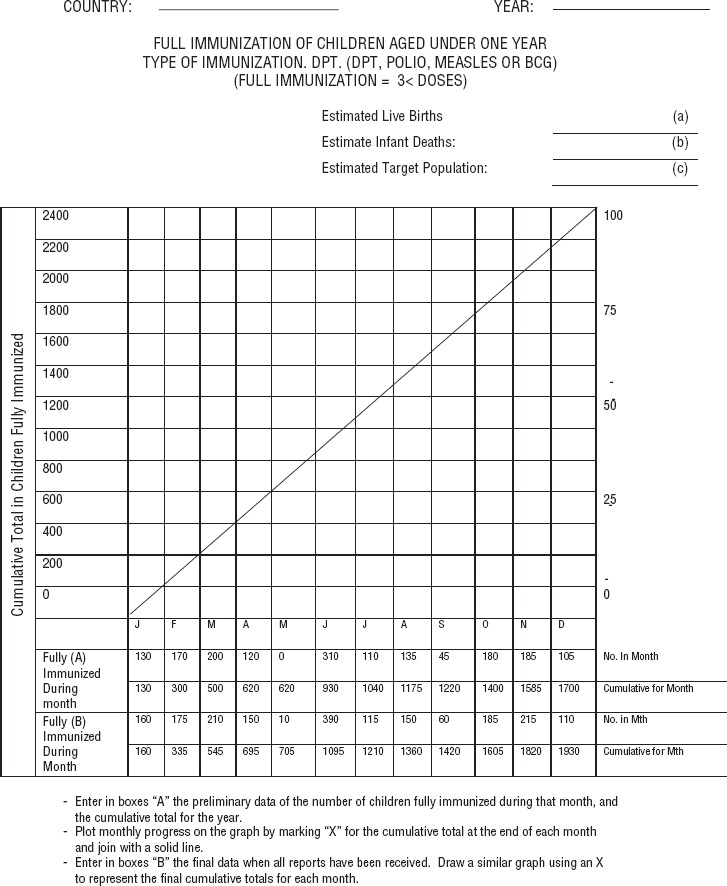
Vaccination coverage monitoring chart developed by Henry Smith, first PAHO Immunization Advisor for the English- and Dutch-speaking Caribbean, 1980

***Source:*** EPI program, PAHO.

The first PAHO Immunization Advisor for the English and Dutch-speaking Caribbean (Henry Smith) developed a simple yet effective tool known as the Immunization Coverage Monitoring Chart (Figure 1). This data visualization tool enables immunization managers to 1) set and evaluate monthly goals, based on the target population; 2) compare expected performance with actual coverage over different time periods; 3) monitor coverage of each vaccine by dose; and 4) accurately depict immunization coverage in each health district. The chart was used by managers at all levels (subnational and national) and national coverage data from each country was sent monthly to the Immunization Advisor to allow for aggregation and monitoring of coverage subregion-wide. Through this process, coverage data for individual countries were also monitored monthly and used to determine the level of support provided to national EPI managers to strengthen and improve their programs (9). This chart, which is still being used by all countries/territories in the subregion, was later adopted by WHO for global use (10, 11).

September 1981 was the first annual meeting for the national EPI managers. Currently, all countries/territories of the English- and Dutch-speaking Caribbean as well as Haiti and the French overseas territories^h^ are invited to participate in the meeting. The main objectives of the meeting are to analyze the achievements and challenges of the current year and to plan activities for the upcoming year. Integral to the process is the development of a national Plan of Action for Immunization for each country, with an estimated budget. In addition, meeting participants provide technical updates, country representatives share their experiences and best practices, and training specialists provide instruction designed to address the deficits and weaknesses of the program. Peer exchanges across countries to build capacity and augment succession planning for EPI managers are also an integral part of the subregional program.

With the oversight of the PAHO Immunization Advisor, CAREC has responsibility for providing laboratory and surveillance support for the program.Weekly reporting and monitoring/evaluation of the diseases and syndromes associated with vaccine-preventable diseases continue to be critical strategies to maintain disease control and/or elimination. Special emphasis is placed on the achievement of the elimination standards for the surveillance indicators.

For the first 25 years of the program (until his retirement in 2002), Ciro de Quadros, the former Immunization Chief at PAHO, provided the subregion with strategic leadership, guidance, and facilitated innovation, which helped energize the country teams.

The English- and Dutch-speaking Caribbean has also been instrumental in ensuring the expansion of vaccination benefits population-wide (e.g., actively vaccinating adult risk groups in preparation for and during mass gatherings such as the Cricket World Cup 2007) as well as legislation and awareness (e.g., a subregion-led resolution to the World Health Assembly in 2012 led to World Immunization Week).

Health promotion and education is an ongoing part of the Immunization program and is used to promote the benefits of vaccination, encourage mothers to vaccinate their children at the optimum age, and disseminate information on vaccines. In more than 95% of the countries in the subregion, immunization is required for school entrance, and appropriate policies and legislation have been enacted through the health and/or education sectors. In one of the subregion’s countries, the legislation includes financial and custodial sanctions for noncompliance.

## RESULTS

### Coverage

The establishment of the EPI in the English- and Dutch-speaking Caribbean resulted in focused activities, including national training and the development of operational guidelines. By 1980, all countries and territories were conducting routine immunization with the diphtheria-pertussis-tetanus (DPT), poliomyelitis, measles, and bacille Calmette-Guerin (BCG) vaccines (3). During the period 2002–2006, all countries/territories in the subregion introduced the childhood vaccines for hepatitis B (Hep B) and Haemophilus influenza type b (Hib) as well as the routine second doseof the measles, mumps, and rubella (MMR) vaccine (MMR2).

**FIGURE 2.**
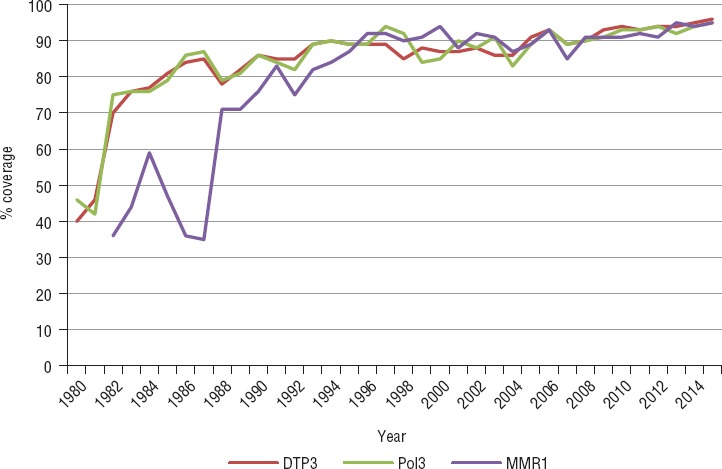
Average coverage by vaccine (third dose of diphtheria-tetanus toxoid and pertussis (DTP3); third dose of oral or inactivated polio (Pol3); and first dose of measles, mumps, and rubella (MMR1)), English- and Dutch-speaking Caribbean, 1980–2015

***Source:*** Country reports to EPI-CAREC and WHO/UNICEF Joint Reporting Forms for Immunization

With consistent technical assistance from PAHO, there was a steady increase in the subregion’s coverage of three doses of DPT (DPT3), from 38% in 1970 to 96% in 2015 (12). Coverage for the primary series for all common antigens in use (DPT3; three doses of Hep B, Hib, and polio; one dose of BCG; and one dose of MMR) was at least 95% in 2015. MMR2 coverage was 90% for the subregion (Figure 2).

As anticipated, the increased vaccination coverage led to a decrease in the incidence of the respective diseases. The last cases of poliomyelitis, indigenous measles, diphtheria, CRS, neonatal tetanus, and rubella in the subregion were, respectively, in 1982, 1991, 1994, 1999, 2000, and 2001.

### Disease elimination

CARICOM’s member states and associate members have kept immunization high on the agenda of health cooperation in the subregion. In 1988, the CARICOM Health Ministers passed a resolution to eliminate measles by 1995. By 1998, CARICOM had passed a similar resolution for rubella and CRS (elimination by the year 2000) (13, 14). The countries in the subregion readily embraced these goals.

**FIGURE 3.**
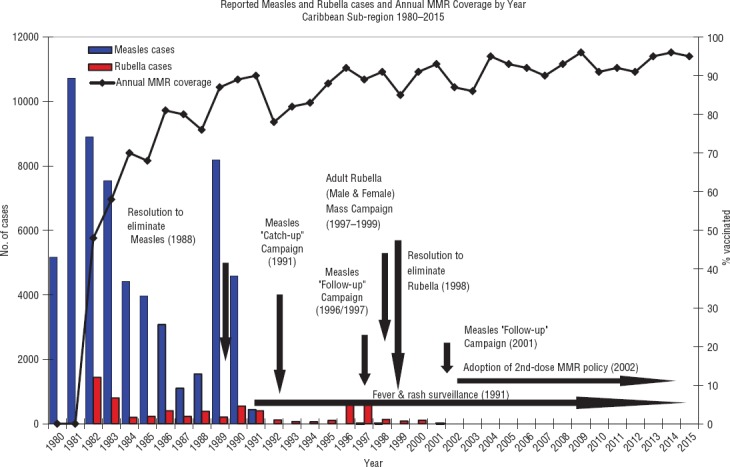
Annual measles, mumps, and rubella (MMR) coverage and reported measles and rubella cases, by year, English- and Dutch-speaking Caribbean, 1980–2015

***Source:*** Rubella and measles cases reported to EPI-CAREC and via country WHO/UNICEF Joint Reporting Forms for Immunization.

The elimination of these diseases was achieved through collective efforts, including coordinated “catch-up” and “follow-up” mass vaccination campaigns, and the strengthening of routine or “keep-up” vaccination of various targeted age groups, including adult males, who were reached at their places of leisure, and work, including the agricultural fields (Figure 3). These efforts were supported by strong active surveillance for suspected cases, using standardized case definitions, in both public and private surveillance sites (including hotels), as well as laboratory diagnosis done centrally at CAREC (15). Training of private-sector health care workers in the principles of surveillance for vaccinepreventable diseases and reporting requirements was also carried out.

Timely investigation of all suspected cases using the “first contact” strategy^i^ and outbreak control measures for confirmed importations also contribute to sustaining elimination of these diseases. This strategy, in collaboration with ongoing public awareness efforts through social mobilization and public education, and the drop in age for administration of the second dose of MMR (to 2 years old) has helped keep the subregion free of indigenous measles (since 1991), CRS (since 1999), and rubella (since 2001).

The diversity of the English- and Dutch-speaking Caribbean in terms of ethnicity, culture, religion, and terrain (which includes hills, rainforests, and plains) combined with its small population facilitated the testing of PAHO’s elimination strategies for measles and rubella within a short timeframe. Lessons learned in the subregion about accessing hard-to-reach populations were refined and replicated in other countries in the Americas, which became the first region in the world to be certified as having eliminated rubella and CRS (in 2015) as well as measles (in 2016).

The English- and Dutch-speaking Caribbean’s risk for reimportation of vaccine-preventable diseases due to the high rate of tourism was underscored with the recording of 1) imported cases of measles in the Bahamas and Trinidad and Tobago in 1997, and in Jamaica in 1998, 2008, and 2011, and 2) an imported case of rubella in Bermuda in 2008. With the exception of one import-related measles case in Ja-maica in 2008, no secondary cases or out-breaks resulted from the imported cases, a good indicator that the subregional EPI has been successful in ensuring high population immunity.

The outbreak of poliomyelitis in Jamaica in 1982, which included 60 cases (77% < 10 years old) and three deaths, resulted in a program advisory to countries/territories in the subregion to ensure improved population immunity and heightened surveillance for the disease. Technical guidance was provided by PAHO’s Immunization Advisor, and laboratory support for testing of samples was provided by CAREC. Since 1982, there have been no more cases of wild or vaccine-associated poliomyelitis in the subregion.

## DISCUSSION

The structure and function of the EPIs in the English- and Dutch-speaking Caribbean were positively influenced by two major decisions. The first was the appointment of a PAHO Immunization Advisor for the subregion, and the second was centralizing the responsibility for the provision of the program’s laboratory and surveillance support at CAREC.

The annual meeting of the English- and Dutch-speaking Caribbean EPI managers has been a crucial component for building and strengthening the program in the subregion. The meeting, now held in November of each year, is rotated from country to country, facilitating the participation of other health care workers from the host country involved in immunization and primary health care, who benefit from the technical updates. The face-to-face meetings allow for candid discussion among the managers about their program experiences, including innovative strategies/solutions, and challenges. Joint agreement on and planning of key activities for the upcoming year, including campaigns and coordinated approaches to the subregion’s goals, such as the introduction of the inactivated polio and human papilloma virus (HPV) vaccines and the switch from the trivalent to the bivalent oral polio vaccine, are some of the positive actions realized through the annual meeting. Relationships established and strengthened throughout the year help provide support for managers, strengthen the program, and facilitate the provision of vaccines to/from countries experiencing over/undersupply to prevent wastage or stockouts. The use of social media platforms (e.g., *WhatsApp)* for sharing information, strategies, motivational messages, and encouragement in times of natural disasters or other hardship underscores the feeling of program solidarity.

The use of specific strategies to motivate the health team and national EPI managers include incentives such as annual awards (plaques/trophies and certificates) for achievements in surveillance and immunization coverage. Observation visits, participation in EPI evaluations in the countries, and dissemination of technical and educational materials help build capacity, especially for new EPI managers and surveillance officers. Succession planning for EPI managers (identifying candidates to assume responsibility pending retirements, promotions, or attrition) is also encouraged, and the chosen successor is invited to attend the EPI managers’ meeting with the outgoing manager. This helps to introduce the new manager to the group and facilitate a smooth transition and transfer of responsibility for the program.

Development of templates for policy documents and procedural manuals are coordinated by PAHO’s subregional Immunization advisor and shared with the countries for use in the development of their own national policies and manuals. These are revised on a regular basis.

Strong collaboration with ministries of education and children’s services in the English- and Dutch-speaking Caribbean has also contributed to the success of the EPI program. Through legislation in public health and education portfolios, more than 95% of countries in the subregion have mandatory vaccination requirements for school entry. This facilitates high vaccination coverage for children under 5 years old and promotes joint or synergistic activities for advocacy and social mobilization. Support for vaccination has also been strengthened through early partnerships with the United Nations Children’s Fund (UNICEF), Rotary International, Child Fund (formerly the Christian Children’s Fund), and the Canadian Public Health Agency (for technical, financial, and other support), and collaboration with medical and nursing associations and the faith-based community, especially for introduction of newer vaccines such as the conjugated pneumococcal and HPV vaccines.

To promote immunization as an integral component of universal health care, all countries in the English- and Dutchspeaking Caribbean integrated vaccination in their primary health care systems and avoided the establishment of a vertical program. In addition, Vaccination Week in the Americas is used by about 50% of countries in the subregion to provide integrated services such as deworming, nutrition assessment, and counseling, as well as screening for chronic noncommunicable diseases.

The dedication of the health care workers, who often go beyond the call of duty to ensure that the Immunization program is implemented, deserves recognition and respect. Without them, the sustainability of the EPI program would be a challenge. Efforts are therefore made to keep them abreast of the latest developments in the field of immunization through in-country annual training workshops and the dissemination of educational materials and newsletters.

The Immunization program in the English- and Dutch-speaking Caribbean has contributed to improved health outcomes for infants and children. The absence of vaccine-preventable diseases from the top leading causes of childhood mortality reflects the progress that has been made.

## CONCLUSIONS

The English- and Dutch-speaking Caribbean is proud of its achievements and leadership with respect to the Immunization program, which has been a model for other programs. However, the countries/territories in the subregion recognize that there is more to be done. While national coverage is relatively high, there is a certain amount of inequity in some countries/territories, as 5%–7% of communities across the subregion have coverage below 80%. However, the commitment remains strong for achieving at least 95% coverage for all vaccines in all municipalities and a world free of vaccinepreventable diseases. The ongoing high-quality technical support from PAHO has been well appreciated and continues to be relied on by the countries/territories of the subregion.

## Acknowledgments

The authors wish to acknowledge the governments and National EPI managers of the English- and Dutch-speaking Caribbean countries over the years.

## Disclaimer

Authors hold sole responsibility for the views expressed in the manuscript, which may not necessarily reflect the opinion or policy of the RPSP/PAJPH or the Pan American Health Organization (PAHO).

## Footnotes

a Anguilla, Antigua and Barbuda, Aruba, Bahamas, Barbados, Belize, Bermuda, British Virgin Islands, Cayman Islands, Curaçao, Dominica, Grenada, Guyana, Jamaica, Montserrat, Netherlands Antilles, St. Kitts and Nevis, Sint Maarten, St. Lucia, St. Vincent and the Grenadines, Suriname, Trinidad and Tobago, and Turks and Caicos Islands.

b Unpublished reports of annual Caribbean EPI Managers’ meetings, PAHO (1982-2016).

c http://www.gavi.org

d Member countries: Antigua and Barbuda, Bahamas, Barbados, Belize, Dominica, Grenada, Guyana, Haiti, Jamaica, Montserrat, St. Kitts and Nevis, St. Lucia, St. Vincent and the Grenadines, Suriname, Trinidad and Tobago. Associate members: Anguilla, Bermuda, British Virgin Islands, Cayman Islands, and Turks and Caicos Islands. Aruba, Colombia, Curaçao, Dominican Republic, Mexico, Puerto Rico, Sint Maarten, and Venezuela have observer status.

e All member/associate member countries listed above for CARICOM, with the exception of Haiti and the addition of Aruba and Netherlands Antilles. At the time, CAREC (which became the Caribbean Public Health Agency (CARPHA) in 2013) was administered by PAHO on behalf of its then 21 member countries.

f Heads of government and various councils of government ministers working on priority agendas for the Caribbean Community, such as human and social development.

g From Guyana (South America) at latitude 2°N to Bermuda at latitude 32°N and Suriname at longitude 54°W to Belize at longitude 89°W.

h Overseas departments French Guiana, Guadeloupe, and Martinique.

i Collection of all information and relevant laboratory samples during the first contact the patient makes with the health system.
